# High-risk type-2 diabetes mellitus patients, without prior ischemic events, have normal blood platelet functionality profiles: a cross-sectional study

**DOI:** 10.1186/s12933-015-0244-x

**Published:** 2015-06-12

**Authors:** Gadi Shlomai, Tal Haran-Appel, Tal Sella, Yoni Grossman, Hagit Hauschner, Nurit Rosenberg, Ehud Grossman

**Affiliations:** Department of Internal Medicine D and Hypertension Unit, The Chaim Sheba Medical Center, Derech Sheba 1, Tel Hashomer, Ramat-Gan 52621 Israel; The Dr. Pinchas Borenstein Talpiot Medical Leadership Program 2013, Tel-Aviv, Israel; Sackler Faculty of Medicine, Tel-Aviv University, Tel-Aviv, Israel; Department of Oncology, Chaim Sheba Medical Center, Tel Hashomer, Israel; Institute of Thrombosis and Hemostasis, Chaim Sheba Medical Center, Tel Hashomer, Israel

**Keywords:** Diabetes mellitus, Platelets activity, Primary prevention, Cardiovascular risk, Aspirin

## Abstract

**Background:**

Patients with type 2 diabetes mellitus (DM) display a predisposition for vascular disease. Platelets taken from vasculopathic diabetic patients, show enhanced stimuli-induced activation and aggregation responses. Aspirin remains the cornerstone antiplatelet agent for secondary prevention of vascular complications among diabetic patients, yet evidence of its efficacy and safety in primary prevention are conflicting. Our aim was to assess whether high risk diabetic patients, without previous ischemic events, have abnormal platelet functionality profiles.

**Methods:**

The study included 82 diabetic patients and 86 matched non-diabetic patients without prior ischemic events nor treatment with anti-platelet medications. Blood samples were analyzed for platelet markers of activation, turnover and leukocyte-platelet interactions.

**Results:**

Our final analysis included 122 males (74 %), with a mean age of 61 years. Mean platelet volume (MPV) was similar between the diabetic patients and controls (9.2 fL for both). Following activation, PAC-1 binding and P-selectin expression were found comparable between the diabetic patients and controls (83 % versus 81 % and 76 % versus 74 %, respectively). Leukocyte-platelet aggregates (LPAs) were similar between the diabetic patients and controls (18 % versus 17 %, respectively). Neutrophil-platelet aggregates (NPAs) and monocyte-platelet aggregates (MPAs) were also found similar in the diabetic patients and controls. Elevated fasting plasma glucose was associated with increased LPAs rates.

**Conclusions:**

High risk type-2 diabetes mellitus patients, without prior ischemic events, have normal blood platelet functionality profiles.

## Background

Patients with type 2 diabetes mellitus (DM) display a predisposition for accelerated atherosclerosis, manifested as a 2–4 fold increased risk for premature cardiovascular (CV) disease [1]. Blood platelets play a pivotal role in the blood clotting process by mediating the primary phase of hemostasis. Their involvement in atherogenesis and thrombotic complications has been previously well documented [2, 3]. Platelets taken from diabetic patients, particularly those with vascular instability and angiopathy, have been found to have increased baseline activation levels as well as enhanced stimuli-induced activation and aggregation responses (platelet hyper-reactivity) [4]. The pathophysiology underlying the dysregulated signaling pathways of platelets in DM is multifactorial and associated with the pernicious effects of persistent hyperglycemia [5–10], an abnormal lipid profile [11–13], insulin resistance [14–16] and oxidative stress [17, 18].

Diabetic patients present with persistent thromboxane-dependent platelet activation, with aspirin playing a pivotal role in the prevention of vascular complications in DM [19]. Nonetheless, while aspirin remains the cornerstone anti- platelet agent for secondary prevention of vascular complications among all patients, irrespective of DM status [20], direct evidence of its efficacy and safety in primary prevention is lacking or at best inconclusive [21–24].

In order to resolve discrepancies among the results obtained in different clinical trials, several meta-analyses were conducted. However, these studies demonstrated that primary prevention with aspirin generates only a modest beneficiary effect on CV risk reduction [25–29]. Recently, the American Diabetes Association (ADA) published guidelines stating that prophylactic aspirin therapy appears to have a modest effect on ischemic vascular events with an absolute decrease in events, depending on the underlying CV risk [30].

The 2014 European Society of Cardiology (ESC)/European Association for the Study of Diabetes (EASD) guidelines also do not recommend primary prevention with aspirin for low risk patients [31]. Nevertheless, the ADA recommended that primary prophylaxis with aspirin therapy for diabetic patients is indicated for those who are at an increased cardiovascular risk (10-year risk >10 %), which includes most men aged >50 years or women aged >60 years who have at least one additional major risk factor [30]. These recommendations essentially support aspirin primary prophylaxis for the majority of diabetic patients.

Real-life data regarding platelet activity and reactivity in high CV risk diabetic patients without previous CV events, are limited and conflicting. Herein, we evaluate whether high risk diabetic patients without previous ischemic events and not treated with anti-platelet medications, have abnormal platelet functionality profiles.

## Methods

### Subjects

**Fig. 1:**
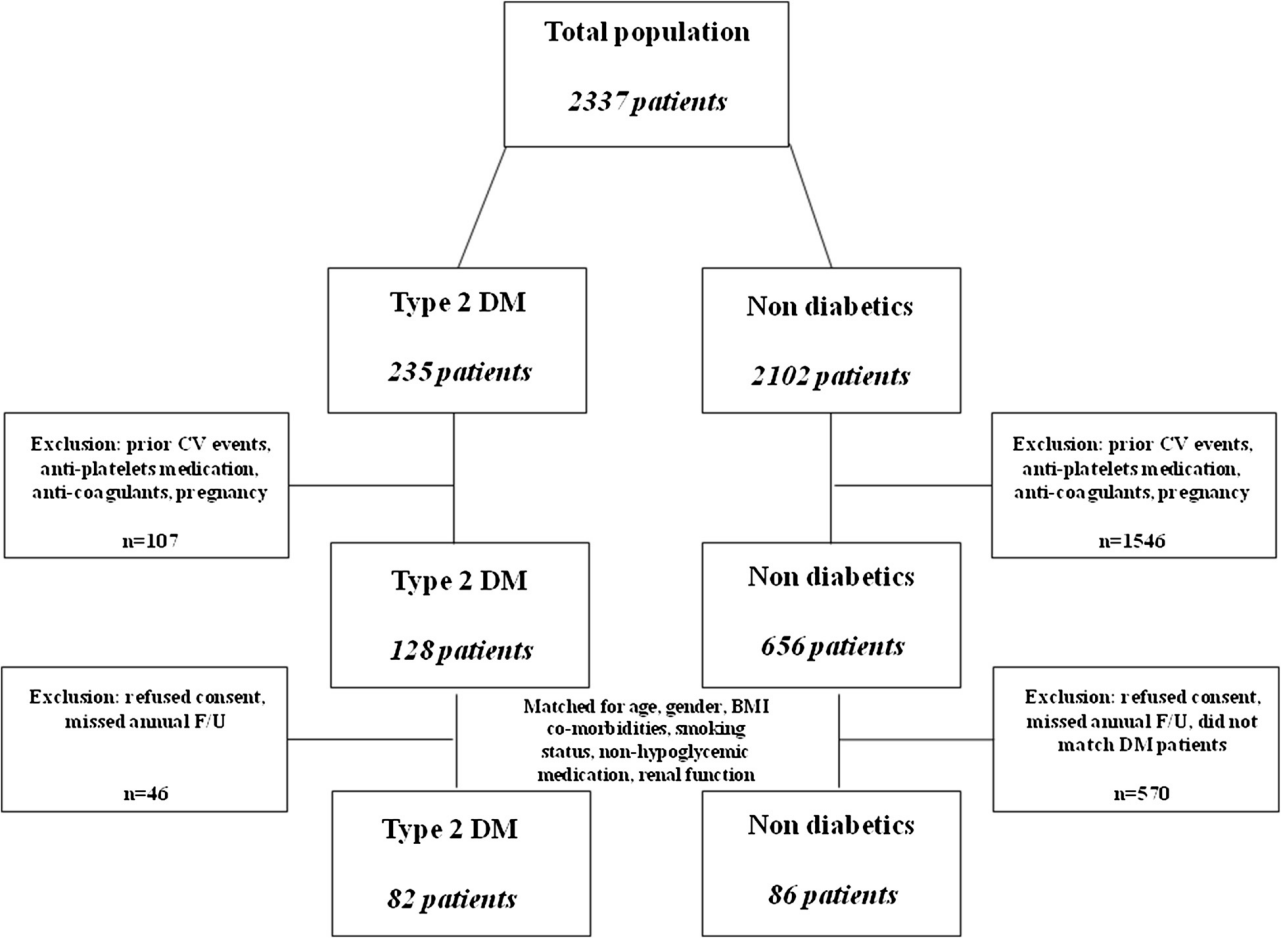
Two thousand thirty seven patients from the “Institute for Medical Screening of the Chaim Sheba Medical Center” were screened. Of them 235 patients had type 2 diabetes mellitus (DM) and 1202 were not diabetic. Patients were excluded if they had prior CV events or treated with any anti-platelets medications or anticoagulants. One hundred twenty eight DM patients and 656 non diabetic patients were eligible for inclusion. After further exclusion due to absence from annual F/U or refusal to consent the study population comprised of 82 DM patients and 86 matched non-diabetic control subjects. *Abbreviations: DM type 2 diabetes mellitus, *CV* Cardiovascular, *F/U* Follow up, *BMI* Body mass index

Subjects included were examined at the Institute for Medical Screening, Chaim Sheba Medical Center, Israel between June 2014 and January 2015. Out of 2337 scheduled subjects, we identified 235 diabetic patients and 2102 non-diabetics patients. Patients were excluded if they had had a past medical history of CV disease or had been treated with any anti-platelet medications or anticoagulants. Pregnant women were also excluded. Out of 235 diabetic patients, 107 were excluded and 46 refused consent or did not appear for their scheduled annual examination. Out of 2102 non-diabetic patients, 1546 were excluded and 570 patients refused consent, did not appear for their scheduled annual examination or did not match the diabetic patients. Subsequently, the study group included 82 diabetic patients and 86 non-diabetic subjects matched for age, gender, body mass index (BMI), co morbidities, smoking status, medications and renal functions (Fig. 1).

The study protocol was approved by the local institutional review board and complies with the Helsinki declaration. Written consent was given by all subjects.

### Definitions

Diabetes mellitus was defined when either a diagnosis of DM was documented in the medical chart; when fasting plasma glucose was >126 mg/dL (7.0 mmol/L) on two separate readings; the hemoglobin A1C (HbA1C) level was ≥6.5 % (47.5 mmol/mol) or when the subject had taken insulin or oral hypoglycemic medications.

Hypertension was defined when either two separate blood pressure (BP) readings were ≥140 mmHg for systolic BP and/or ≥90 mmHg for diastolic BP; a history of hypertension was reported or when the subject had taken antihypertensive medications. Hyperlipidemia was defined when the diagnosis was documented in the medical chart or when the patient had been chronically prescribed statins or fibrates. Smoking status was determined according to a questionnaire where participants were divided into current or non-smokers. BMI was calculated from the height and weight parameters measured on the day of inclusion.

Information regarding prescribed medications was obtained from documented the medical charts. All information regarding clinical diagnoses and medical therapy was cross referenced and confirmed with the patients upon signing of the informed consent form.

### Protocol

Upon consent, venous blood was drawn for a complete blood count, renal function tests, serum electrolytes, serum fasting glucose, HbA1C level, a fasting lipid profile, mean platelet volume (MPV) and platelet functionality assays.

CV risk assessment was conducted according to the “General Cardiovascular Risk Profile of the Framingham Heart Study” [32]. This cardiovascular risk score calculator was utilized for its inclusion of diabetic patients.

### Platelet functionality assays

Venous blood was drawn into a 3.2 % sodium citrate blood-collecting tube. Whole blood was diluted fivefold by adding a Ca^2+^-free HEPES buffer (145 mM NaCl, 5 mM KCl, 1 mM MgSO4, 0.5 mM NaH2PO4, 5 mM glucose, and10mM Hepes/Na; Sigma) within 30 min of collection. Diluted blood samples were incubated for 10 min at room temperature with PC5-conjugated anti-CD45 mAb (BD Bioscience, San Jose, CA) and FITC-conjugated anti-CD41 mAb (Beckman Coulter, Marseille, France) for leukocyte-platelet aggregate analysis. Next, samples were fixed with 1 % paraformaldehyde (Electron Microscopy Sciences, Hatfield, PA) for 10 min at room temperature, followed by a dilution of 4.6-fold with distilled water for red cell lysis and sample dilution. Samples were analyzed by flow cytometry [33]. For detection of platelet activation, blood samples were centrifuged for 10 min at 800 rpm in preparation for platelet rich plasma (PRP). The PRP were diluted tenfold with PBS and incubated for 20 min at room temperature with an anti PE- conjugated anti CD62p monoclonal antibody (BioLegend, San-Diago, CA) and a FITC- conjugated PAC-1 monoclonal antibody (Becton Dickinson. San Jose, CA), specific to the active conformation of integrin αIIbβ3 in the presence or absence of 10 μM ADP (molab) as a platelet activator. Samples were diluted fivefold with PBS and analyzed by flow cytometry.

### Statistical analysis

For the univariate analysis, percentages were calculated for categorical variables and means with standard deviations, for continuous variables. The chi-square test for categorical variables and unpaired *t*-test for continuous variables were performed to determine significant differences in baseline characteristics and platelet functions between diabetic patients and controls. Multiple linear regression was performed to assess predictors of platelet function.

Models were adjusted for age, gender, HbA1C, fasting plasma glucose (FPG), low density lipoprotein (LDL), high density lipoprotein (HDL), total cholesterol, hemoglobin, platelet count, estimated glomerular filtration rate (eGFR) and Framingham risk score (FRS). All p value calculations were two-tailed and considered statistically significant if their value was <0.05. Statistical analyses were performed by the IBM SPSS version 20.0 (Chicago, Illinois, USA).

## Results

### Participants’ characteristics

The study included 82 diabetic patients (74 % males) with a mean age of 62 ± 8 years. The controls (*n* = 86) were matched for age, gender, BMI, smoking status, renal function, CV related co-morbidities and non-hypoglycemic medications (Table 1). Compared to the controls, diabetic patients had higher fasting plasma glucose levels (*p < 0.001*), elevated HbA1C levels (*p < 0.001*), a higher CV risk as represented by FRS (*p < 0.001*), and lower HDL (*p = 0.04)*, LDL (*p < 0.001)* and total cholesterol levels (*p < 0.001)* (Table 1).

**Table 1:** Basic characteristics

Characteristic	Cases (DM)	Controls	*P* value
*n*	82	86	
Gender (male) – *n* (%)	61 (74)	61 (74)	0.615
Age (y) – mean (SD)	62 (±8)	62 (±7)	0.941
BMI (kg/m2) – mean (SD)	28 (±4)	27 (±4)	0.12
Active smoking – *n* (%)	4 (5)	6 (7)	0.747
FRS – mean (SD)	26 (±13)	16 (±9)	<0.001
Comorbidities – *n* (%)			
Hypertension	45 (55)	47 (55)	0.976
Hyperlipidemia	61 (74)	62 (72)	0.737
Hypoglycemic medications – *n* (%)			
Biguanides	58 (70)	N/A	
GLP1-RA	3 (4)	N/A	
DPP-4 antagonists	25 (30)	N/A	
SU/Meglitinides	10 (12)	N/A	
Insulin	8 (10)	N/A	
Medications – *n* (%)			
Beta blockers	16 (20)	12 (14)	0.334
Calcium channel blockers	14 (17)	17 (20)	0.653
ACEi/ARB	33 (40)	31 (36)	0.575
Diuretics	4 (5)	9 (10)	0.175
Statins	53 (65)	46 (54)	0.142
Laboratory values – mean (SD)			
Hemoglobin (g/dl)	14 (±1)	14 (±1)	0.635
Platelets (K/microL)	222 (±54)	216 (±52)	0.401
Fasting plasma glucose (mg/dl)	136 (±29)	91 (±8)	<0.001
HbA1c (%) (mmol/mol)	6.7 (±0.9) (49.7)	5.4 (±0.3) (35.5)	<0.001
LDL (mg/dl)	109 (±24)	121 (±24)	<0.001
HDL (mg/dl)	46 (±14)	50 (±12)	0.040
Total Cholesterol (mg/dl)	162 (±30)	179 (±31)	<0.001
eGFR (mL/min/1.73 m^2)^	79 (±19)	76 (±15)	0.289

*DM* type 2 diabetes mellitus, *BMI* Body Mass Index, *FRS* Framingham Risk Score, *GLP-1RA* Glucagon Like Peptide-1 Receptor Agonist, *DPP-4* Dipeptidyl Peptidase-4, *SU* Sulfanilurea; *ACEi* Angiotensin Converting Enzyme Inhibitor, *ARB* Angiotensin Receptor Blockers, *HbA1C* Hemoglobin A1C, *LDL* Low Density Lipoprotein, *HDL* High Density Lipoprotein, *eGFR* Estimated Glomerular Filtration Rate

### Platelet functionality assays

MPVs were similar between diabetic patients and controls (9.2 ± 1.3 fL for both, *p = 0.9)*.

**Fig. 2:**
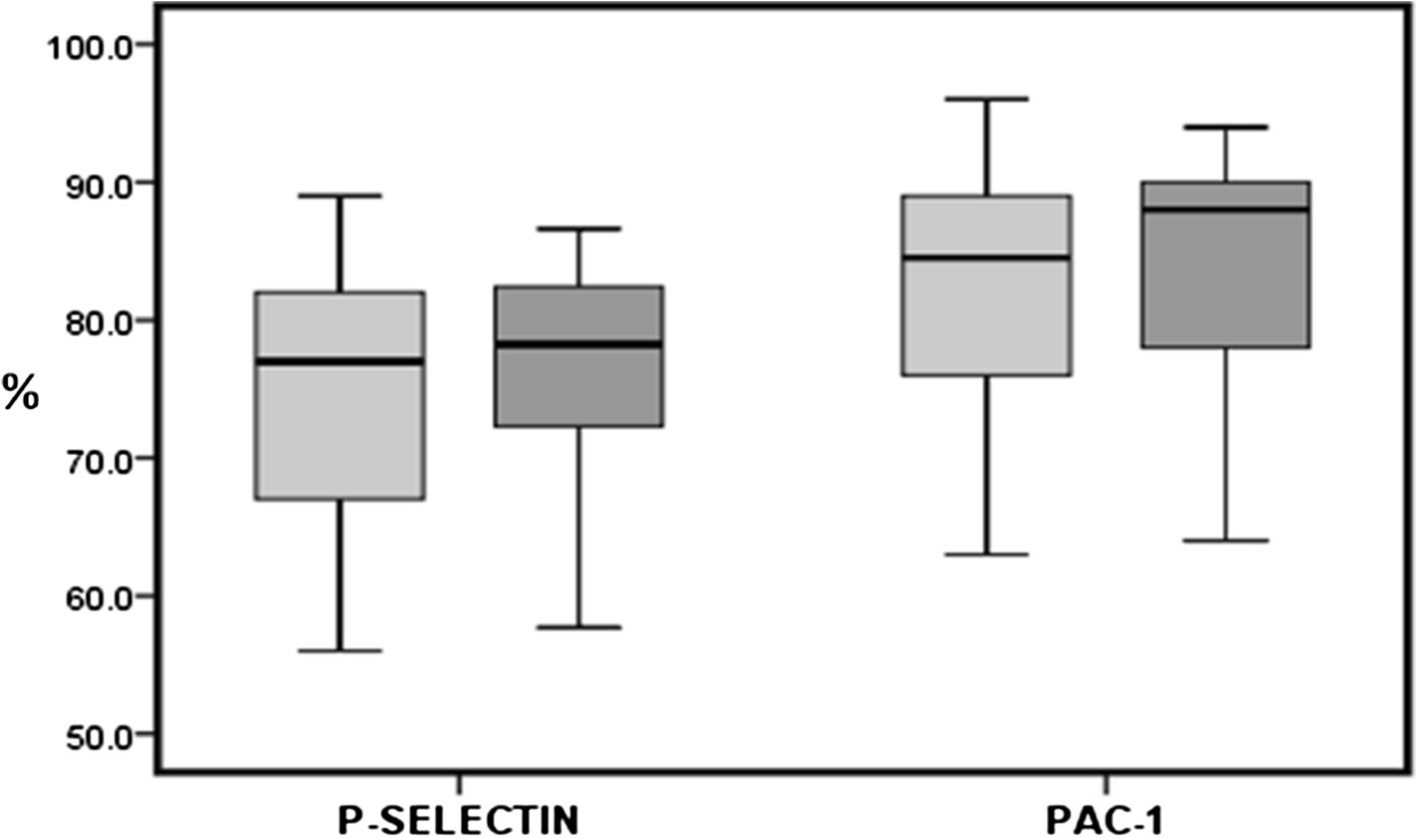
Percentage of cells positive for PAC-1and P-selectin expression following ADP activation. DM patients are represented in *dark gray* (*right box*) and non-diabetic matched controls are represented in *light gray* (*left box*). *Abbreviations: DM type 2 diabetes mellitus

Following ADP activation, the percentage of cells positive for PAC-1 binding and P-selectin expression, markers for fibrinogen receptor activity and α-granule secretion, respectively, were comparable between diabetic patients and controls (83 % for diabetic patients versus 81 % for controls, *p = 0.17*, and 76 % for diabetic patients versus 74 % for controls, *p = 0.14*, respectively) (Fig. 2). Similarly, calculating the mean fluorescence intensity (MFI) following ADP activation also demonstrated comparable results between groups (an 11.9- fold increase in diabetic patients versus an 11.6- fold increase in non-diabetic patients and a 13.3- fold increase for diabetic patients versus a 13.8- fold increase in non-diabetic patients, respectively, data not shown). Moreover, the MFI of PAC-1 and P-selectin for resting platelets was also similar (10.67 ± 10.99 in diabetic patients versus 9.09 ± 8.2 in non-diabetic patients and 11.38 ± 8.98 in diabetic patients versus 9.86 ± 7.4 in non-diabetic patients, respectively, data not shown). These results indicate that both the baseline platelet activation and the ADP-induced activation response were similar for both diabetic patients and controls.

**Fig. 3:**
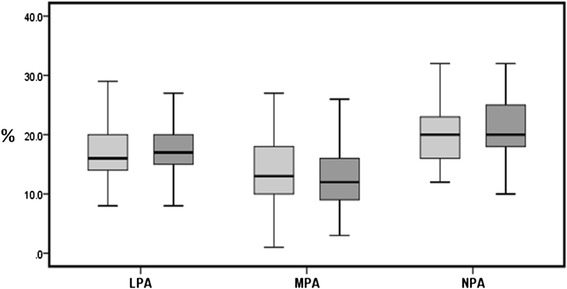
Percentage of LPAs, MPAs and NPAs following ADP activation. DM patients are represented in *dark gray* (*right box*) and non-diabetic matched controls are represented in *light gray* (*left box*). *Abbreviations: LPAs Leukocyte-platelet aggregates, *MPA* Monocyte-platelet aggregates, *NPAs* Neutrophil-platelet aggregated, *DM* type 2 diabetes mellitus

A similar total of leukocyte-platelet aggregates (LPAs) in diabetic patients and non-diabetic patients (18 % versus 17 %, respectively, *p = 0.25*) were found in the leukocyte aggregation assays. Neutrophil-platelet aggregates (NPAs) and monocyte-platelet aggregates (MPAs) were also found to be similar (14 % in diabetic patients versus 15 % in non-diabetic patients, *p = 0.68*, and 22 % in diabetic patients versus 21 % in non-diabetic patients, *p = 0.36*, respectively) (Fig. 3).

### Effects of glycemic burden and cardiovascular risk on platelets functionality

The multivariate linear regression model demonstrated that elevated fasting plasma glucose was associated with increased LPAs (*p = 0.01*). However, neither HbA1C levels nor FRS were associated with any changes in LPAs rates (Table 2). Effects of a glycemic burden or elevated CV risk scores on the percentage of cells positive for PAC-1and P-selectin expression were not found (data not shown).

**Table 2:** Results of multiple linear regression analysis (Dependent variable: percentage of LPAs increase)

Covariates	B Coefficient (SE)	Partial correlation	*P*
Age	−0.09 (0.07)	−0.15	0.235
Gender	−0.24 (1.3)	−0.02	0.854
HbA1C	−0.45 (0.49)	−0.10	0.361
FPG	0.04 (0.01)	0.28	0.013
LDL	0.01 (0.04)	0.04	0.852
HDL	0.08 (0.04)	0.25	0.082
FRS	0.02 (0.05)	0.06	0.654

*LPAs* Leukocytes-platelets aggregates, *PAC-1* Procaspase activating compound, *HbA1c* Glycated hemoglobin, *FPG* Fasting plasma glucose, *LDL* Low density lipoprotein, *HDL* High density lipoprotein, *eGFR* Estimated glomerular filtration rate, *FRS* Framingham Risk Score, *HbA1C* Hemoglobin A1C, *FPG* Fasting Plasma Glucose

## Discussion

In this study, we compared blood platelet functionality profiles of high CV risk diabetic patients, without prior CV events, to those of matched controls. We found that diabetic patients and matched controls display comparable levels of platelet markers of activation, turnover and leukocyte-platelet interactions.

In the multivariate analysis, an association was found between elevated fasting plasma glucose levels, albeit not HbA1C values or FRS, with an increase of LPAs rates. No association was found between the other activation and aggregation markers with fasting blood glucose, HbA1C levels or FRS. Currently, data regarding the importance of a glycemic burden on platelets activity are conflicting. There are several studies, which demonstrated the importance of intensive glycemic control [34–36] while others have found no effect of intensive glucose control on platelets activity [37].

Several studies have previously demonstrated that platelets taken from diabetic patients show aberrant platelets functionality profiles. However, in most of these trials, platelet hyper-reactivity was found predominantly in patients with either a very high glycemic burden [7, 37, 38] or documented clinically significant vasculopathy [4, 37, 39–42], or to a lesser extent in diabetic patients without angiopathy [34–36, 40, 42, 43]. Conversely, our study population uniquely comprised high CV risk well-controlled diabetic patients without prior ischemic events untreated with any anti-platelet medication. Although a few studies have included patients with similar characteristics, they differ significantly from our analysis in several aspects such as primarily focusing on either aspirin resistance among diabetic patients [43] or assessing platelet morphological characteristics in pre-diabetic patients [35]. In addition, these studies assessed platelets functionality by either the impedance method through whole-blood aggregometry [34], which might be confounded by various blood components or by light transmission aggregometry (LTA) using platelet rich plasma [43], which is a good assay for evaluation of anti-platelets drug response or bleeding tendency, but not for assessing hyperaggregation.

In our study, however, we assessed platelet activity markers such as P-selectin and PAC-1, as well as leukocyte-platelet aggregates. We tested both activation markers before and after adding ADP. We then evaluated both baseline activation level and the ADP-induced activation response.

Low-dose aspirin remains the cornerstone of anti-platelet therapy for secondary prevention of coronary artery disease, cerebrovascular disease and mortality in diabetic and non-diabetic patients with established vascular disease. However, although its role in the primary prevention of CV events is controversial, many physicians prescribe prophylactic aspirin therapy for their high CV risk patients, specifically diabetic patients.

During the last 10 years, several large scale prospective randomized controlled trials have been conducted, assessing the beneficiary effect of aspirin as a primary prevention measure among high risk diabetic patients. The Early Treatment Diabetic Retinopathy Study (EDTR) revealed a significant decrease in the relative risk of myocardial infarction at 5 years in patients with type 1 and type 2 DM, without any benefit in mortality rates [21]. The Prevention of Progression of Arterial Disease and Diabetes (POPADAD) did not indicate any benefit of aspirin or antioxidants in the primary prevention of CV events [22]. In the Japanese Primary Prevention of Atherosclerosis with Aspirin for Diabetes (JPAD), primary prevention with aspirin did not reduce the rates of all CV events, yet the rates of fatal coronary and cerebrovascular events, a secondary end point, were reduced [23]. Furthermore, the recently published data of the Japanese Primary Prevention Project (JPPP) demonstrated that primary prevention with aspirin does not reduce the risk of CV morbidity and mortality in high CV risk patients, including those with DM [24].

In addition, several other trials in which diabetic patients constituted only subgroups within broader trials of aspirin prophylaxis, yielded conflicting results [44–49]. Furthermore meta-analyses demonstrated that primary prevention with aspirin produces only a modest beneficiary effect on CV risk reduction [25–29]. Currently, both ADA guidelines and ESC/EASD guidelines recommend (level C) prophylactic aspirin therapy for high CV risk (10-year risk > 10 %) diabetic patients [30, 31], which includes most men aged >50 years or women aged >60 years who have at least one additional major risk factor (family history of CV disease, hypertension, smoking, dyslipidemia, or albuminuria) [30]. Thus, these recommendations essentially support the use of aspirin as a primary prophylaxis for the majority of diabetic patients.

The results of our study do not support these recommendations, as our multivariate analysis did not detect an association between elevated Framingham Risk Scores and platelet markers of reactivity. Thus, herein, we present novel data suggesting that well-controlled diabetic patients without prior ischemic events have normal platelet functionality profiles, regardless of their CV risk.

The main limitation of our study is that CV risk factors assessed in our population were well-controlled. The enrolled diabetic patients had a low glycemic burden reflected by fasting blood glucose and HbA1C. They also showed minimal aberrancies in lipid metabolism with relatively low smoking rates. Nevertheless, although somewhat limited to a well-controlled diabetic patient population, our results have merit. Diabetic patients whose CV risk factors are well-controlled constitute a large proportion of the diabetic population, with a majority still being widely prescribed unnecessary and potentially hazardous medications such as aspirin [50].

## Conclusions

In conclusion, we demonstrated that in well-controlled high CV risk diabetic patients without prior angiopathy and no anti-aggregate treatment, platelet functionality profiles are normal and comparable to matched controls. Furthermore, for the most part, glycemic burden and CV risk were not found associated with increased expression of platelet markers of hyper-reactivity. Our findings and recent results of large scale clinical trials collectively undermine the role granted for aspirin as a primary prevention measure in high CV risk diabetes patients.
